# Costs and outcomes of active and passive case detection for visceral leishmaniasis (Kala-Azar) to inform elimination strategies in Bihar, India

**DOI:** 10.1371/journal.pntd.0009129

**Published:** 2021-02-03

**Authors:** Natalie J. Dial, Graham F. Medley, Simon L. Croft, Tanmay Mahapatra, Khushbu Priyamvada, Bikas Sinha, Lucy Palmer, Fern Terris-Prestholt

**Affiliations:** 1 Department of Global Health and Development, Faculty of Public Health and Policy, London School of Hygiene and Tropical Medicine, London, United Kingdom; 2 Faculty of Infectious and Tropical Diseases, London School of Hygiene and Tropical Medicine, London, United Kingdom; 3 CARE India Solutions for Sustainable Development, Patna, Bihar, India; 4 Mott MacDonald, London, United Kingdom; Universidade Federal de Minas Gerais, BRAZIL

## Abstract

**Background:**

Effective case identification strategies are fundamental to capturing the remaining visceral leishmaniasis (VL) cases in India. To inform government strategies to reach and sustain elimination benchmarks, this study presents costs of active- and passive- case detection (ACD and PCD) strategies used in India’s most VL-endemic state, Bihar, with a focus on programme outcomes stratified by district-level incidence.

**Methods:**

Expenditure analysis was complemented by onsite micro-costing to compare the cost of PCD in hospitals alongside index case-based ACD and a combination of blanket (house-to-house) and camp ACD from January to December 2018. From the provider’s perspective, a cost analysis evaluated the overall programme cost of each activity, the cost per case detected, and the cost of scaling up ACD.

**Results:**

During 2018, index case-based ACD, blanket and camp ACD, and PCD reported 1,497, 131, and 1,983 VL-positive cases at a unit cost of $522.81, $4,186.81, and $246.79, respectively. In high endemic districts, more VL cases were identified through PCD while in meso- and low-endemic districts more cases were identified through ACD. The cost of scaling up ACD to identify 3,000 additional cases ranged from $1.6–4 million, depending on the extent to which blanket and camp ACD was relied upon.

**Conclusion:**

Cost per VL test conducted (rather than VL-positive case identified) may be a better metric estimating unit costs to scale up ACD in Bihar. As more VL cases were identified in meso-and low-endemic districts through ACD than PCD, health authorities in India should consider bolstering ACD in these areas. Blanket and camp ACD identified fewer cases at a higher unit cost than index case-based ACD. However, the value of detecting additional VL cases early outweighs long-term costs for reaching and sustaining VL elimination benchmarks in India.

## Author summary

Visceral leishmaniasis (VL) is targeted for elimination in India by 2020, where early identification and prompt treatment are essential measures for reaching and sustaining incidence benchmarks. Both active- and passive- case detection (ACD and PCD) strategies have been employed in recent years, and evaluating the cost and outputs of each is now important for sustaining funding and political momentum. This study presents overall and unit costs for PCD, index case-based ACD (where neighbours in the vicinity of a recent VL case are screened), and a combination of blanket and camp ACD (involving house-to-house case searching and weekly diagnostic camps) in Bihar, India during 2018. Results of this study indicate that a larger proportion of VL cases were found through PCD in high incidence districts, which may be related to increased interaction with ACD officers. ACD can be bolstered in meso- and low-incidence districts where educational exposure to VL is low and risk of resurgence is high. Although blanket and camp ACD unit costs were at least four times higher than index case-based ACD, the number of VL cases identified through this approach may warrant the investment to achieve VL elimination. Cost and outcomes of VL case finding approaches need to be continuously evaluated until elimination benchmarks are reached and integrated into sustained surveillance programmes.

## Introduction

### The Kala-Azar elimination programme (KEP)

Visceral leishmaniasis (also known as Kala-Azar) is a parasitic Neglected Tropical Disease (NTD) endemic in 83 countries worldwide, with reported global incidence just over 17,000 in 2018 [1]. Transmitted by the female phlebotomine sand fly, VL is characterised by prolonged fever, enlarged spleen and liver, anaemia, substantial weight loss, and a 95% fatality rate if left untreated [2]. International elimination efforts alongside increased availability of rapid diagnostic tests and improved treatment have contributed to a substantial decline in VL cases over the past decade, particularly in the Indian Subcontinent (ISC) [1, 3]. Due to elusive transmission dynamics confounded by asymptomatic carriers and the sequela post-Kala-Azar dermal leishmaniasis (PKDL), VL is currently targeted for elimination as a public health problem (EPHP), signifying sustained control activities are essential for reaching and maintaining incidence targets to prevent disease resurgence [4].

Over 25% of the global VL burden exists in India, where 85% of cases are reported in the state of Bihar. In 2005, a regional Kala-Azar Elimination Programme (KEP) developed within the ISC to mobilise national programming, international financial support, and drug donations; this has helped facilitate Nepal and Bangladesh achieve EPHP benchmarks [5]. Early case detection is a prioritized measure for reducing transmission in India, which relies on effective surveillance to support accurate diagnosis and complete treatment at the hospital level [6, 7].

However, institutional and socio-economic barriers challenge the integration of rural populations into India’s health system, where patients may consult uncertified rural practitioners (URPs), lack accessibility to and trust in government health facilities, or not seek medical support [8–10]. To expedite identification and referral of potential VL cases, the KEP has supported village-level active case detection (ACD) over the past decade.

### Active and passive case detection (ACD & PCD) in the KEP

Prompt diagnosis and treatment of VL serves two purposes: to prevent death, and to reduce transmission. Through the systematic screening of populations in endemic areas by health staff to find cases, ACD has been shown to contribute to early VL detection in India [6, 8, 11]. Four distinct, but not mutually exclusive, approaches to ACD exist: blanket (house-to-house screening), camp (mobile diagnostic teams visiting targeted villages), index case-based (searching for new cases in the vicinity of confirmed cases) and incentives-based (village health workers paid to find suspected cases) [6]. Although ACD activities are conducted at the village level, suspected cases are then reported at the block level (a sub-district region where elimination targets are measured) as well as within Bihar’s 33 endemic districts.

In Bihar, a range of ACD strategies have been managed by two different organisations: CARE and KalaCORE. Since 2017, the non-profit organisation CARE has worked with the state government to lead index case-based ACD in support of the KEP. Their programme relies on Kala-Azar Block Coordinators (KBCs) conducting snowball-surveillance in the vicinity of recent VL patients at one-, six- and 12- months post-treatment. Fortnightly ACD is then conducted in each village for 12 months after its last reported VL case. Additionally, key informants such as family members, school teachers, and shop keepers, as well as Accredited Social Health Activists (ASHAs), are trained to report potential VL cases to KBCs by telephone. Separately, KalaCORE (a consortium funded by UK Aid) operated from 2015–2019 employing a combination of blanket and camp ACD. KalaCORE recruited and trained KBCs, local medical staff, and other ACD officers to conduct blanket (house-to-house) surveillance in villages with high VL incidence, followed by weekly diagnostic camps to confirm VL infections and refer cases to treatment facilities.

Without active intervention, VL cases are presumed to be found through Passive Case Detection (PCD), that is, symptomatic individuals presenting at a static hospital or Primary Health Centre (PHC) with no prior interaction with a KBC or ACD officer. In 2014, as part of India’s KEP, over 120 VL treatment centres were strategically established within PHCs in close proximity to high endemic villages (with more than five cases per 10,000 population per year) [12]. These treatment centres offer free rK-39 rapid VL diagnostics and single-dose AmBisome treatment through the Government of India’s National Vector Borne Disease Control Programme (NVBDCP), and are designated referral sites for all suspected cases [13].

### Informing and updating EPHP strategies

To achieve Bihar’s EPHP target of less than one VL case per 10,000 population per block per year by 2020, policy-relevant research is needed to assess current VL surveillance strategies [13, 14]. Economic evaluation compares resources required, cases identified, and costs of parallel surveillance programmes for informing priority setting across elimination strategies. Three economic analyses of ACD on the ISC were conducted between 2010–2012, where the cost per case detected through blanket and camp ACD varied between $21-$629 and PCD was excluded as it was considered an integrated public health service [6, 8, 11]. As VL incidence was five-fold higher during that time [1], it is important now to re-evaluate the cost and investment in ACD during a period when numbers of VL cases are low and the elimination target is close.

Importantly, as disease cases decrease often so does financial and political support to maintain control activities given competing disease priorities in lower-middle income countries (LMIC) [15, 16]. Donor-funded VL programmes in India are especially vulnerable at the point around EPHP, where viability, value, and longevity of current programmes must be evaluated to adapt and advocate financial support [17]. Methods for assessing disease screening during EPHP often compare programme costs to the likelihood of reaching elimination benchmarks as a means to provide translatable evidence for decision makers [18–21]. Therefore, the aim of this study was to conduct a cost-minimisation analysis of current VL case detection programmes to determine the least costly approach to achieving EPHP in the endemic state of Bihar.

Results of this study should extend insight into how the health officials could scale-up and modify ACD strategies to reach EPHP efficiently, which may provide generalizable lessons for VL globally as well as other NTDs approaching elimination [22, 23]. This evaluation may also provide information for the eventual integration of VL case detection horizontally into other febrile illness programmes after elimination benchmarks are achieved.

## Methods

### Ethics statement

Ethical approval was obtained from the London School of Hygiene and Tropical Medicine in November 2019 (Reference Number 17763), and permission to collect data from CARE and KalaCORE was subsequently confirmed.

### Cost model

National guidelines for economic analysis in India are not available, where this study followed the Global Health Cost Consortium Reference Case alongside costing literature for disease surveillance in low- and middle-income contexts [24–27].

**Fig 1:**
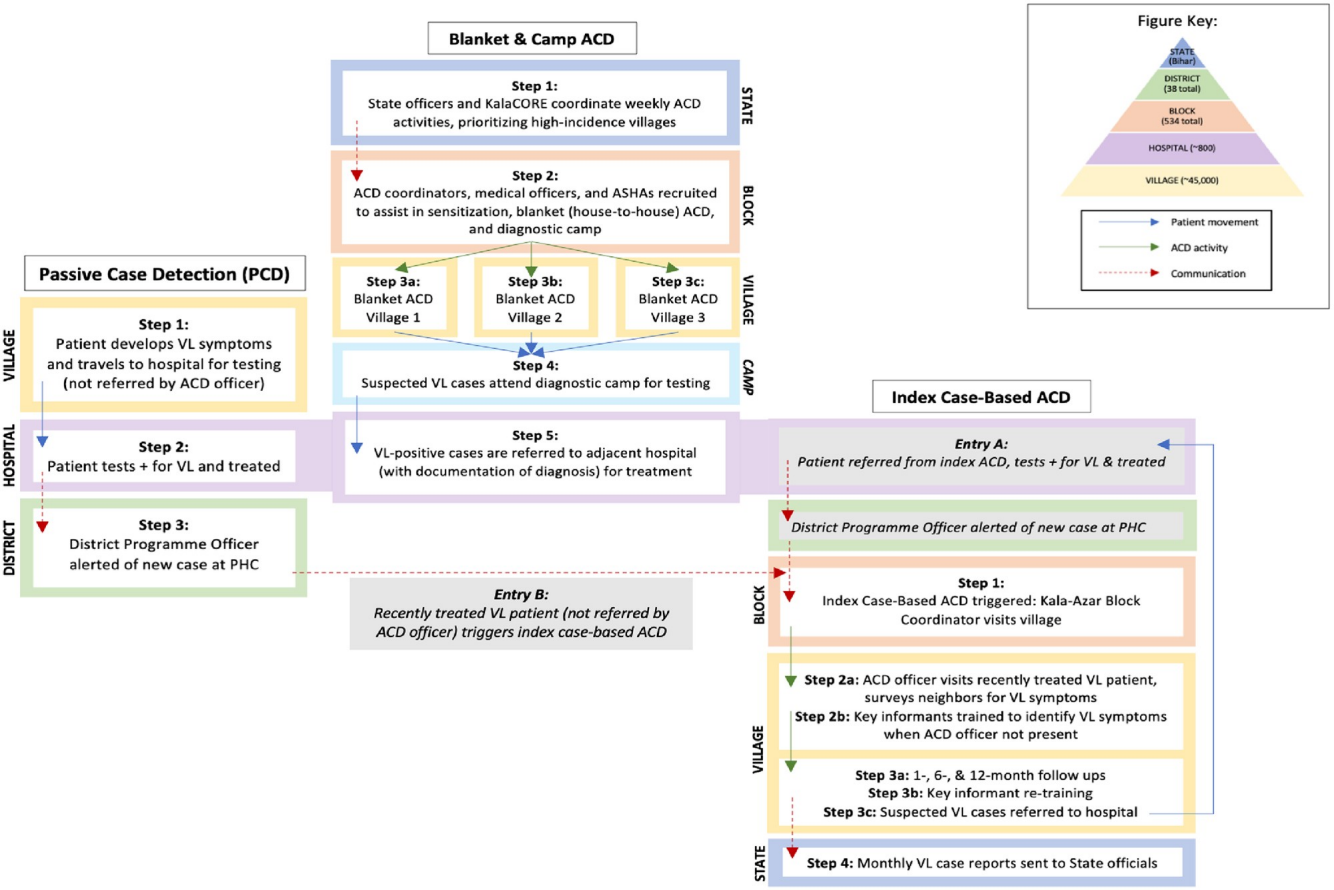
Diagram of VL active and passive case detection in Bihar. Activities, communication, and patient flow involved in PCD, blanket and camp ACD, and index case-based ACD for visceral leishmaniasis in Bihar, India during 2018.

Fig 1 illustrates the basic activities, communication, and patient flow of each programme: PCD, blanket and camp ACD, and index case-based ACD. This study involved both primary and secondary data collection from three organisations and two government facilities involved in ACD and PCD in Bihar, India. Using a top-down approach (including facility-level financial data) supplemented by bottom-up micro-costing (relying on observation and interviewing), programme inputs, costs, and outputs were estimated to generate a cost model from the provider’s perspective (Bihar Ministry of Health and Family Welfare). Given VL seasonality and occurrence of overlapping ACD activities, a full year (January-December 2018) was used to capture transmission dynamics, start-up, and project costs at a standard discount rate of 3%. Costs of treatment were excluded from this study under the assumption that all VL cases are eventually confirmed in hospital and treated same day for all approaches. Although PKDL case finding is integral to the KEP, it was excluded due to lack of comparable data across ACD and PCD programmes.

### Study area

With a population of 104 million, the north-eastern state of Bihar is India’s fifth most impoverished municipality [28]. As Bihar is 89% rural, over 60% of the workforce engages in agricultural and farming activities [28, 29]. One third of the state reportedly lives below the poverty line, accounting for the second highest malnutrition rates in India [29]. Less than 25% of Bihar residents have completed secondary education, where females exhibit the lowest national levels of both educational attainment and labour force participation [29].

### Data sources

#### ACD

Data was collected from CARE regarding index case-based ACD, and from KalaCORE regarding blanket (house-to-house surveillance) and diagnostic camp ACD. Project accounts and expenditure reports from 2018 were collated to determine start-up costs (training, materials, per diems), capital costs (buildings, equipment, vehicles), and recurrent costs (salaries, medical supplies, travel). Economic costs were estimated for donated goods, indirectly purchased equipment, and training. Interviews with financial officers, programme managers, KBCs, and District Programme Officers then triangulated programme reports by examining programme structure, length of activities, and missing costs associated with ACD. Some inputs were also estimated from other VL costing studies in the literature, manufacturer costs for vehicles, rental rates for equivalent office space in Bihar, and market prices of relevant supplies (see S1 Appendix for sources of each input).

Outputs were reported individually by CARE and KalaCORE, detailing the number of suspected, tested, and VL-positive cases identified at the district level through respective ACD programmes during 2018.

#### PCD

The cost of PCD was calculated at the PHC level, designating 2015 as the ‘start-up’ period when VL treatment centres were established in Bihar that included updated diagnostic and treatment protocol. The start-up (training, staff, materials) and capital costs (cold-chain storage, buildings, vehicles) were estimated from programme asset registers and interviews. Recurrent costs to screen and test individual patients were estimated through direct observation and interviews with medical officers to determine staff time for clinical examination, laboratory costs, and prices to stock, maintain and use VL diagnostics in Bihar. Costs and time associated with medical officers’ absence from normal duties to attending training were included.

Outputs included number of patients tested and identified as VL-positive through PCD, which were estimated from incidence reported by the Kala-Azar Management Information System (KAMIS) and Ministry of Health Management and Information System (HMIS) databases. PCD-specific outputs were estimated by subtracting the number of cases identified through ACD from overall VL incidence reported by KAMIS in Bihar during 2018.

### Cost analysis

The annual incremental economic costs of each programme were estimated for the base year 2018. All resources were accounted for, including donated goods and opportunity costs of unpaid time to attend trainings. Start-up, capital, and recurrent costs were analysed by categorising each line item by input type. The start-up period was defined as all costs incurred during project conception and training prior to implementation (first patient screened). Where provider costs were shared across hospital units (overheads, staff time, laboratory equipment), allocation factors were estimated from direct observation, interviewing, and referencing hospital-based costing literature specific to Northern India [30, 31].

Unit costs were calculated in Excel by dividing each programme’s total cost by the total number of cases suspected, tests conducted, and VL-positive cases identified. All costs were reported in local currency, Indian Rupee (INR), and converted to 2018 $USD using central bank exchange rates [32]. The least costly approach of ACD and PCD was evaluated by comparing total and unit programme costs as well as the cost of scaling up ACD to identify further unreported VL cases. Outcomes of each programme were stratified by district-level (high >200 cases, meso 50–200 cases, and low <50 cases) VL incidence in Bihar to evaluate areas where bolstering ACD may have a greater epidemiological advantage.

### Sensitivity analysis

A sensitivity analysis was conducted to determine the robustness of assumptions used in the cost analysis (see S1 Appendix for list of assumptions). To determine the extent to which variation in values, unmeasured variables, and altering key assumptions led to different interpretations or conclusions, a univariate sensitivity analyses varied vehicle life years (+/- 5 years), operation (+/- 10%), discount rate (+/- 3%), central cost allocation factors (+/- 5%), personnel salaries (+/- 10%), and economic life years of start-up, training, and other capital costs (+/- 2 years).

A scenario analysis also explored the impact on variability of the observed intervention, such as personnel time allocation (+/- 5%), number of VL tests conducted (+/- 10%), and price of rK-39 tests (+/- 50%). A multivariate sensitivity analysis illustrated best- and worst-case scenarios according to variation in the univariate parameters.

### Modelling and scale-up

Marginal costs of scaling up each ACD strategy were estimated through variable costs required to detect one additional VL case or conduct one additional test. The cost of scaling up each ACD programme to varying degrees was compared by calculating marginal cost to conduct an additional 25,000 tests (which would identify an additional 3,000 VL cases). Finally, outcomes of each programme were compared by district-level incidence in Bihar, as reported by the KAMIS database during 2018, to understand how costs were likely to be influenced by incidence.

## Results

### Cost summary

#### Programme outputs

Of the 3,611 VL cases reported in Bihar during 2018, 45% were identified through ACD. PCD, blanket and camp ACD, and index case-based ACD respectively conducted 31,000, 1,945, and 12,261 VL tests and found 1,983, 131, and 1,497 VL-positives during this timeframe (Table 1). The percentage of VL-positives identified relative to tests conducted ranged from 7% in both PCD and blanket and camp ACD to 12% in index case-based ACD.

**Table 1:** Cost Summary and outputs of PCD, blanket & camp ACD, and index case-based ACD for VL during 2018.

Cost Category	PCD		Blanket & Camp ACD		Index Case-Based ACD	
	$USD	%	$USD	%	$USD	%
***Start-up***						
Training	$22,304.51	4.56%	$1,565.54	0.29%	$7,699.88	0.98%
Other Start-up	--	--	$1,058.48	0.19%	$767.07	0.10%
**Total Start-up**	$22,304.51	4.56%	$2,624.02	0.48%	$8,466.95	1.08%
***Capital Costs***						
Building & Storage	$8,961.77	1.83%	$1,311.27	0.24%	$591.90	0.08%
Equipment	$11,611.70	2.37%	$4,350.14	0.79%	$4,157.75	0.53%
Vehicles	$837.97	0.17%	$2,399.51	0.44%	$13,574.20	1.73%
Other Capital Costs	$1,121.69	0.23%	$1,128.25	0.21%	--	--
**Total Capital Costs**	$22,533.13	4.60%	$9,189.17%	1.68%	$18,323.85	2.34%
***Recurrent Costs***						
Personnel	$200,781.30	41.03%	$347,630	63.38%	$536,650.78	68.57%
Supplies	$177,295.91	36.23%	$20,972.75	3.82%	$4,774.75	0.61%
Vehicle Operation & Maintenance	$2,793.04	0.57%	$21,885	3.99%	$181,025	23.13%
Building Operation & Maintenance	$51,151.70	10.45%	$430.23	0.08%	$373.88	0.05%
Recurrent Training	$12,525.23	2.56%	$1,555.52	0.28%	$33,038.30	4.22%
Diagnostic Camps	--	--	$144,185	26.29%	--	--
**Total Recurrent Costs**	$444,547.18	90.84%	$536,658.50	97.85%	$755,862.71	96.58%
**TOTAL ANNUAL COSTS**	$489,384.82	100%	$548,471.69	100%	$782,653.51	100%
**Total Costs without Start-up**	$467,080.31	95.4%	$545,847.67	99.52%	$774,186.56	98.8%
**Units & Costs**	N	Cost per ($USD)	N	Cost per ($USD)	N	Cost per ($USD)
**Suspected or Screened VL Case**	225,000	$2.18	2,212	$247.95	16,459	$47.55
**VL Tests**	31,100	$15.74	1,945	$281.99	12,261	$63.83
**VL Positives**	1,983	$246.79	131	$4,186.81	1,497	$522.81
**VL Tests (Recurrent costs only)**		$14.29		$275.92		$61.65
**VL Positives (Recurrent costs only)**		$224.18		$4,096.63		$504.92

Start-up, capital, recurrent, and total annual costs of PCD, blanket and camp ACD, and index-case based ACD in Bihar during 2018, presented in $USD and as a percentage of total programme cost. Cost per suspected or screened VL case, VL tests conducted, and VL-positive case identified in each programme are included.

#### Total programme costs

Total programme cost calculations included both financial and economic costs, where index case-based ACD was the highest at $782,653.51, and blanket and camp ACD and PCD were similar at $548,471.69 and $489,384.82, respectively. Financial costs for index case-based ACD, blanket and camp ACD, and PCD were $559,019.33, $525,996.80, and $279,260.99. Over 90% of costs were recurrent in all programmes, the majority of which were attributed to personnel salary. Vehicle operation, supplies, and the cost of diagnostic camps within blanket ACD were the second highest overall program expenditures.

#### Unit costs

At $4,186.81 per VL-positive case identified, blanket and camp ACD was eight times higher than index case-based ACD at $522.81. However, blanket and camp ACD was only four times higher than index case-based ACD per VL test conducted, at $281.99 versus $63.83. PCD was $246.79 per VL-positive identified and $15.74 per test conducted. Within each ACD programme, the cost per suspected case and cost per test conducted were similar, indicating accuracy in their respective screening strategies.

### Sensitivity analysis

**Fig 2:**
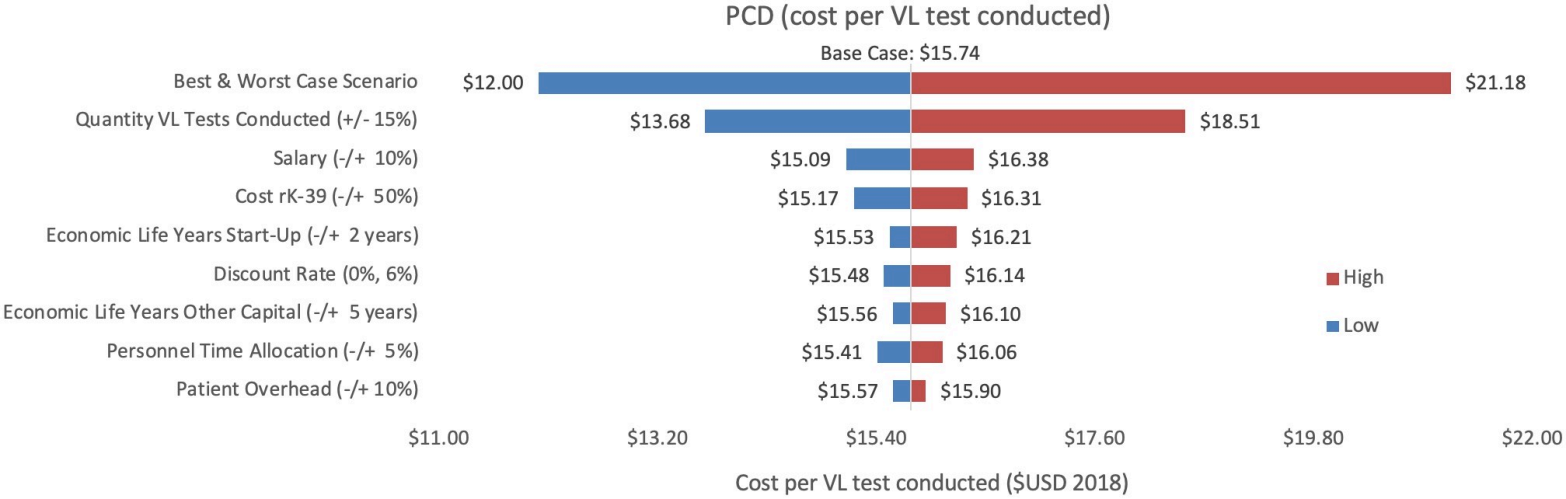
Sensitivity analysis of PCD costs per VL test conducted. Variations in PCD cost per VL test conducted in Bihar during 2018.

**Fig 3:**
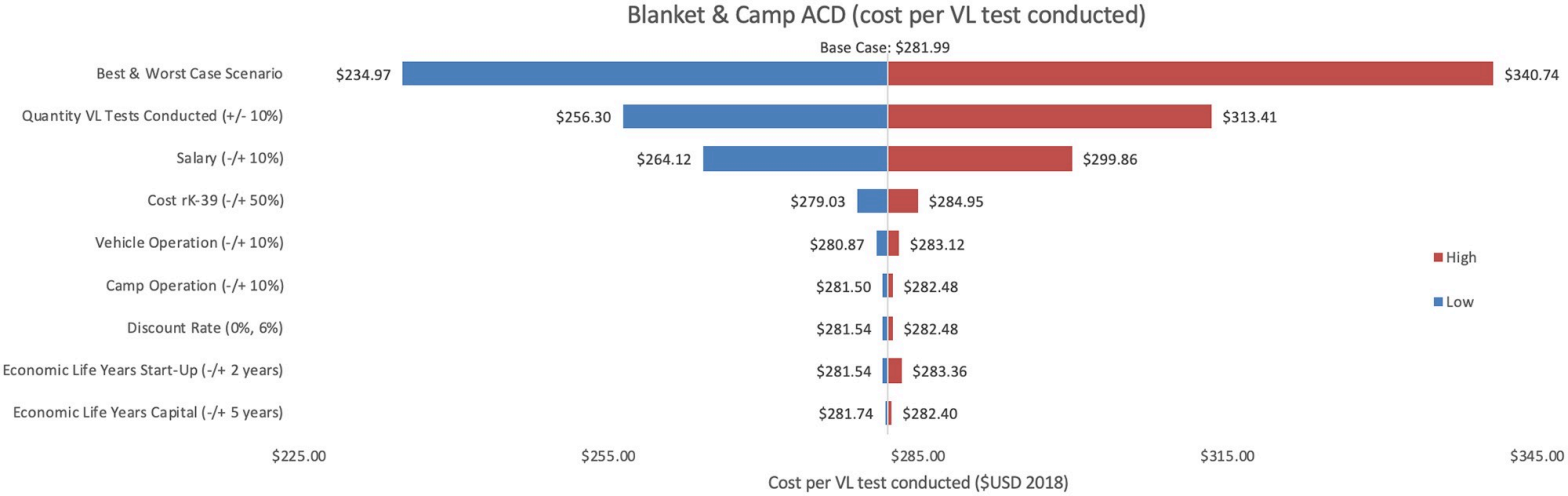
Sensitivity analysis of blanket & camp ACD per VL test conducted. Variations in blanket & camp ACD cost per VL test conducted in Bihar during 2018.

**Fig 4:**
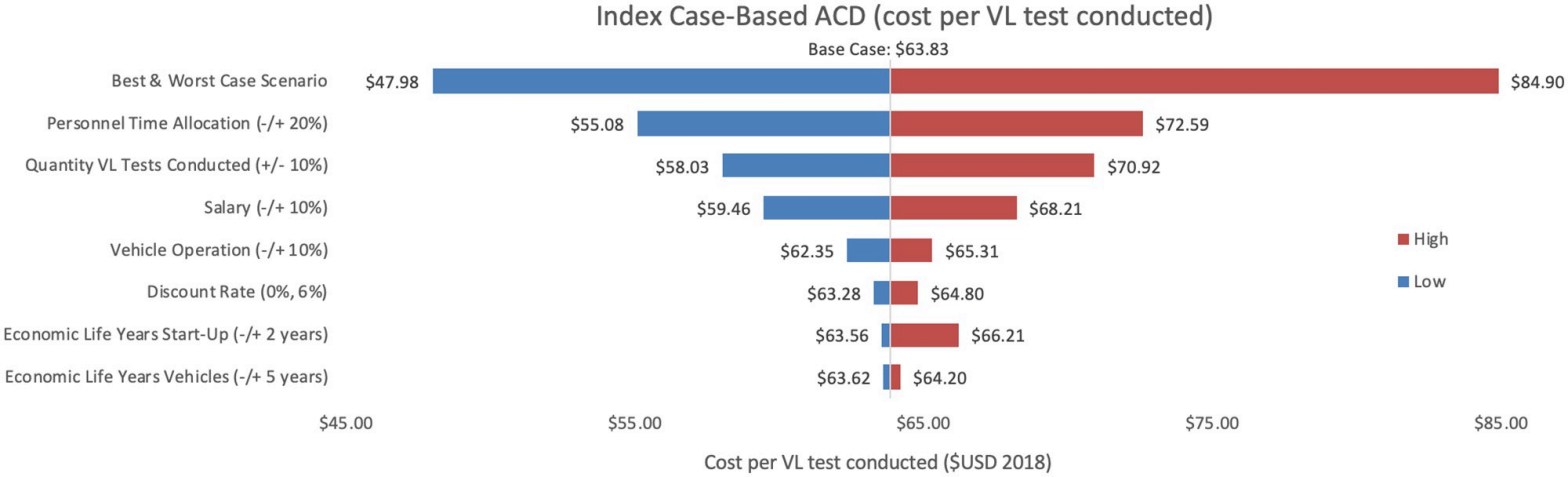
Sensitivity analysis of index case-based ACD per VL test conducted. Variations in index case-based ACD cost per VL test conducted in Bihar during 2018.

**Fig 5:**
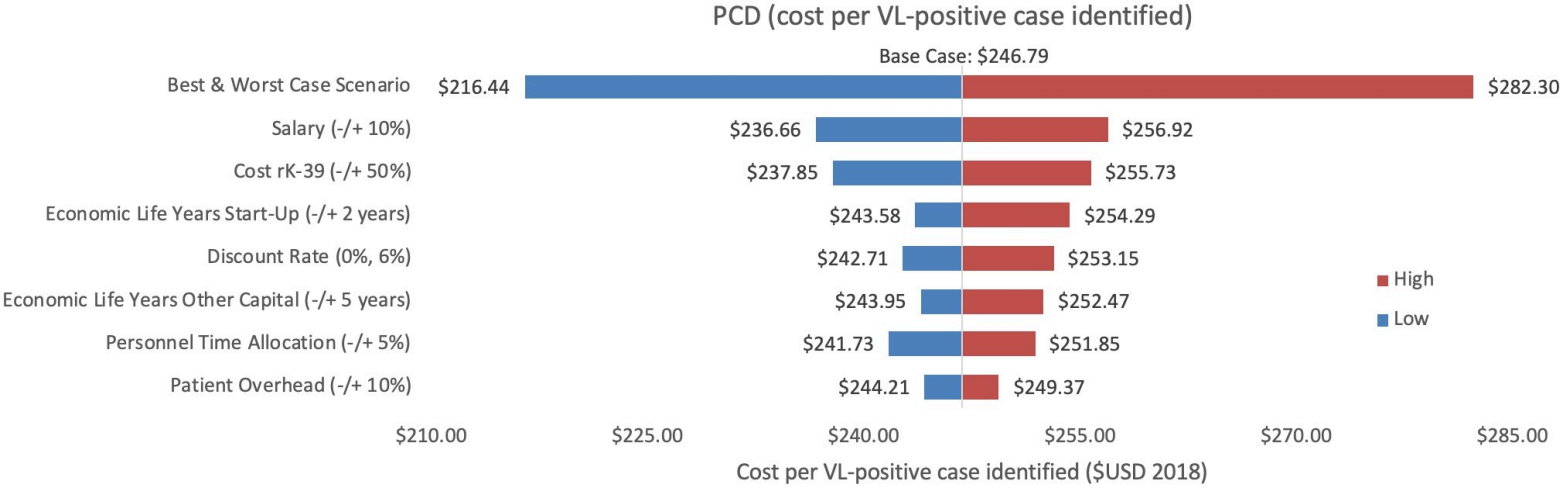
Sensitivity analysis of PCD costs per VL-positive case identified. Variations in PCD cost per VL-positive case identified in Bihar during 2018.

**Fig 6:**
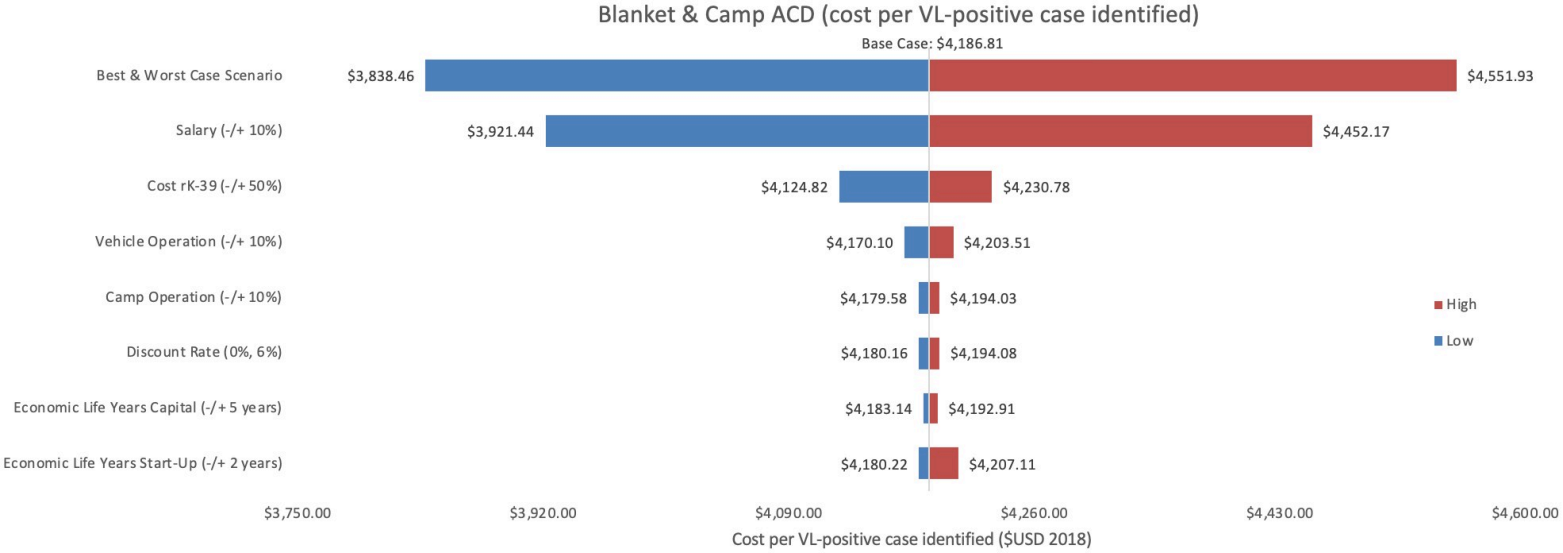
Sensitivity analysis of blanket & camp ACD per VL-positive case identified. Variations in blanket & camp ACD cost per VL-positive case identified in Bihar during 2018.

**Fig 7:**
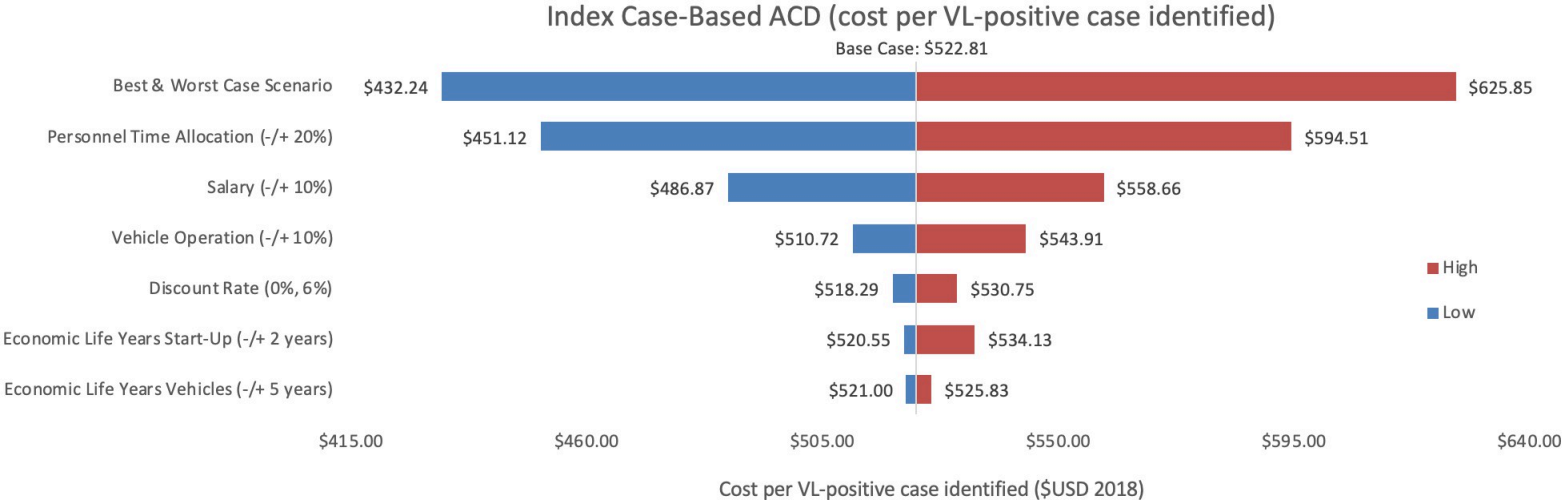
Sensitivity analysis of index case-based ACD per VL-positive case identified. Variations in index case-based ACD cost per VL-positive case identified in Bihar during 2018.

Sensitivity analyses conducted for each of the three programmes are shown, where Figs 2–4 represent variations in cost per VL test conducted and Figs 5–7 represent cost per VL-positive case identified.

#### Cost per VL test conducted

As an alternative scenario, quantity of tests conducted (+/-10%) yielded the largest unit change in cost per test for blanket and camp ACD (ranging from $256.30 to $313.41) and PCD (ranging from $13.68 to $18.51). For index case-based ACD (Figs 4 and 7), increasing or decreasing personnel time allocated to ACD activities contributed the largest unit cost range (from $55.08 to $72.59).

#### Cost per VL-positive identified

As an assumptions test, variations in personnel time and salary produced the greatest influence on cost per case detected in each of the three programmes. Altering staff salaries by 10% would increase or decrease cost per VL case detected between 5–12%.

### District-level incidence

**Fig 8:**
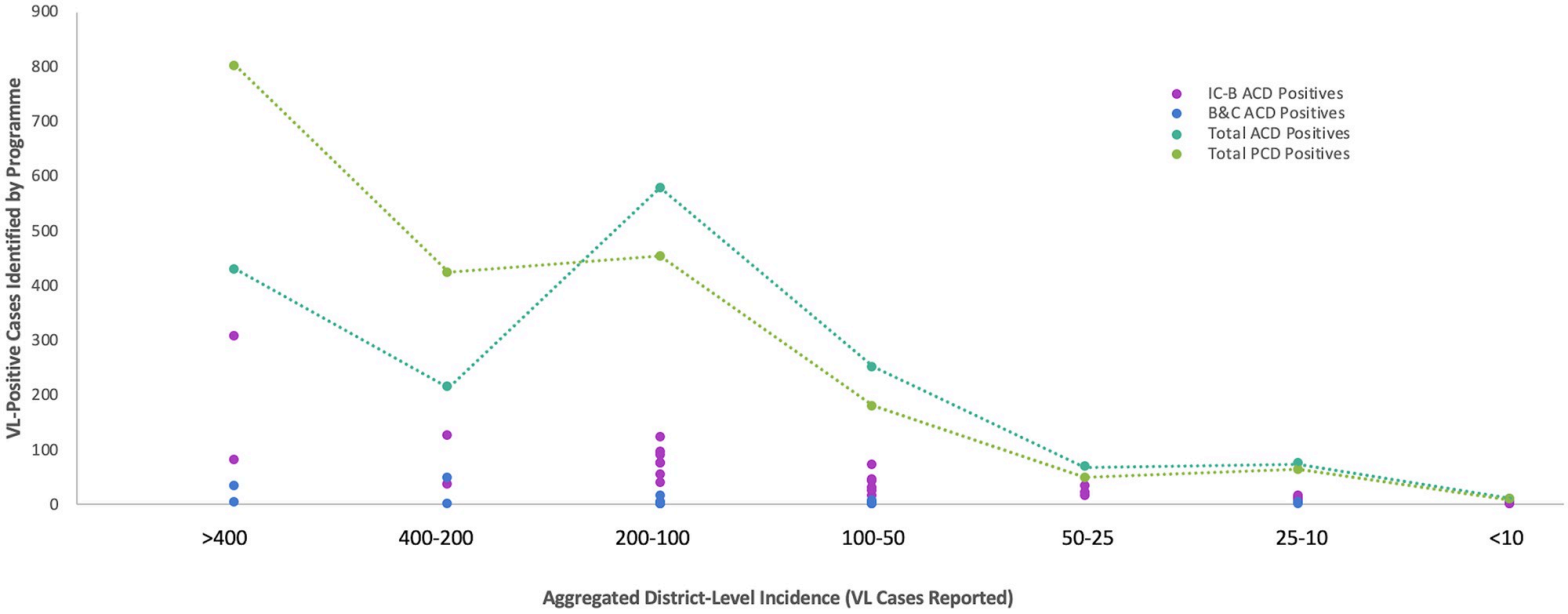
VL-positive cases identified through ACD and PCD stratified by district-level incidence. Number of VL-positive cases identified according to district-level incidence through index case-based ACD (IC-B) (pink), blanket & camp ACD (B&C) (blue), total ACD (index case-based ACD and blanket & camp ACD together) (teal), and PCD (green) in Bihar during 2018.

Within Bihar’s 33 VL-endemic districts, incidence ranged from over 800 to less than 10 cases in 2018. Fig 8 displays the number of district-level VL-positive cases identified within each programme stratified by incidence. PCD identified more VL-positive cases in high incidence districts, while both ACD programmes collectively identified more VL-positive cases across districts reporting less than 200 cases. Blanket and camp ACD mostly targeted ‘hot spots’ and high-incidence villages, but also contributed to detecting some cases in low-incidence districts.

### Cost of scaling up ACD

ACD has the potential to be scaled up to find additional cases, whereas PCD is integrated in the health system without the opportunity to expand existing services. Decision makers may be interested in the costs and outputs of detecting additional VL cases by investing differently in each ACD strategy. To project outcomes of scaling up ACD in Bihar, programme costs of both ACD methods were pooled together as if hypothetically allocated or coordinated through one entity, such as NVBDCP.

**Fig 9:**
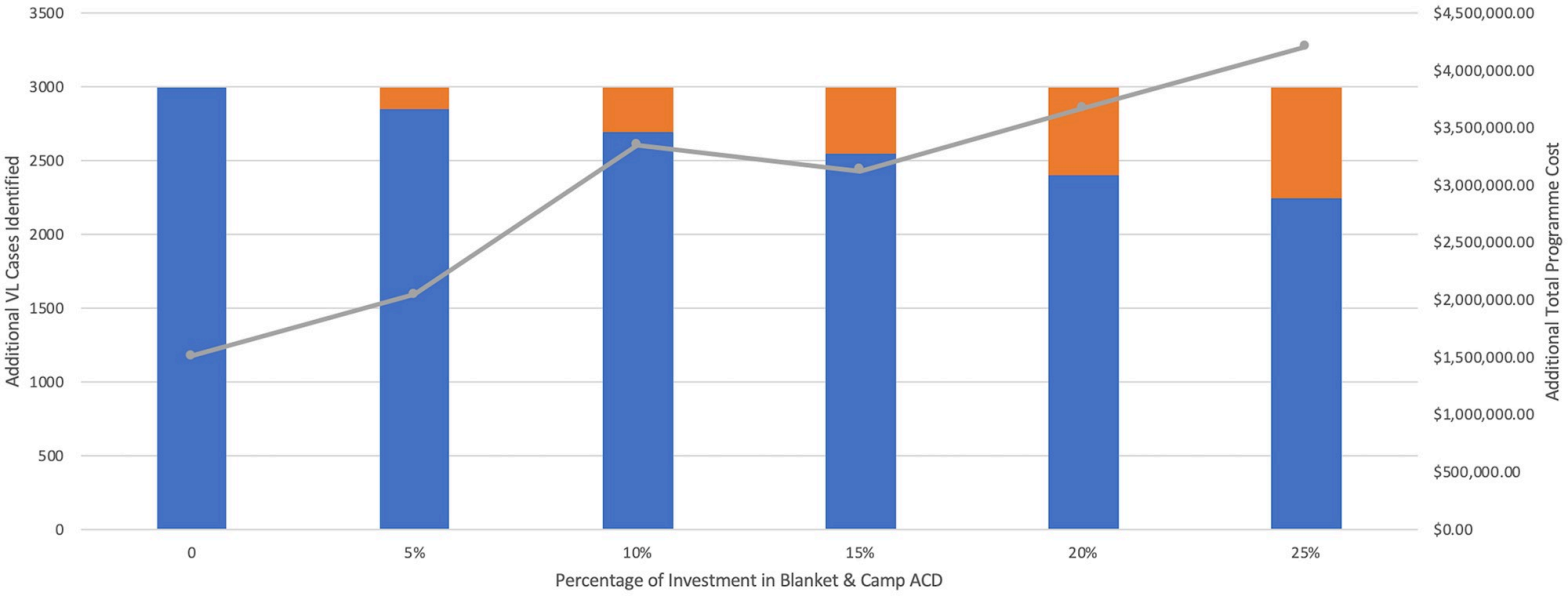
Cost of scaling up ACD to identify additional cases. Cost of scaling up index case-based (IC-B) and blanket & camp (B&C) ACD to identify 3,000 additional VL cases. Total programme cost includes both IC-B and B&C together, as if managed by a single funder.

**Fig 10:**
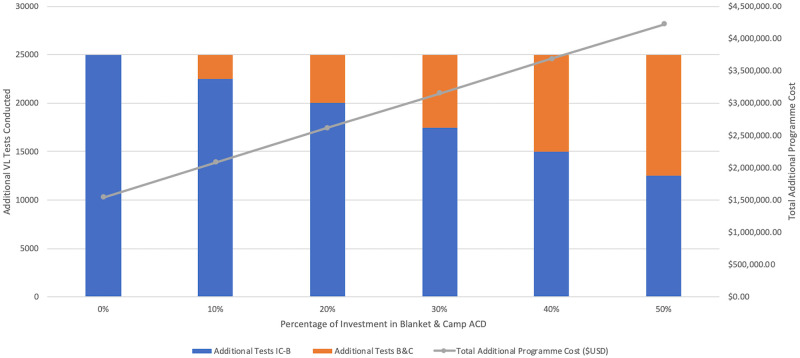
Cost of scaling up ACD to conduct additional tests. Cost of scaling up index case-based (IC-B) and blanket & camp (B&C) ACD to conduct 25,000 additional VL tests. Total programme cost includes both IC-B and B&C together, as if managed by a single funder.

Ninety percent of VL cases identified through ACD in 2018 were detected using the index case-based strategy. WHO declares around one-half of global VL cases are actually reported, therefore Fig 9 displays the cost of scaling up ACD to identify an additional 3,000 VL cases in Bihar [2]. Fig 10 illustrates the additional cost of investing from 0–50% in blanket and camp ACD to conduct 25,000 more VL tests. In each scenario, the overall programme cost would increase from approximately $1,600,000 USD to around $4,000,000 USD if blanket and camp ACD were increasingly relied on.

From the average cost per test conducted of index case-based ($63.83) and blanket and camp ACD ($281.99), the marginal cost per test of scaling up to detect 3,000 additional cases was $61.65 and $275.92, respectively. In both strategies, the marginal cost of scaling up testing was only slightly less than the average cost and continued to decrease minimally with up to 70,000 additional tests conducted.

## Discussion

In addition to evaluating programme and unit costs during 2018, this study revealed that more VL cases were identified through PCD in high-incidence areas and through ACD in meso- and low-incidence areas. This pattern aligns with previous studies on high-incidence areas in the ISC, although there is a lack of literature examining the effects of ACD in meso- and low-incidence populations [6, 8, 11]. The lowest cost for scaling up ACD would solely involve the index case-based strategy given high unit costs of blanket and camp ACD. However, the blanket and camp approach accounted for 8% of VL cases identified through ACD, which may rationalise high unit costs to achieve and sustain EPHP.

Alongside overall and unit costs, evaluating the tactical strengths and weaknesses of each ACD programme informs where to re-direct VL resources in Bihar to reach EPHP. Index case-based ACD is likely more advantageous over time and blanket and camp ACD over space. Effects of index case-based ACD are longer-term as a product of educating key informants to identify and refer potential cases when ACD officers are not present. Blanket and camp ACD is more robust and systematic in a given geographic area, yet house-to-house surveillance is limited by absence of disease symptoms at a given point in time.

### Strengthening educational reach of index case-based ACD

Results of this cost analysis indicate an opportunity to bolster ACD in districts with less than 200 cases, where individuals may have less physical, but also educational, exposure to VL. As the frequency and magnitude of index case-based ACD mirrors VL incidence in each area, high-incidence populations presumably have more interaction with ACD officers and trained key informants. Therefore, these populations might be more likely to recognise VL symptoms early and seek care than lower-incidence populations.

Similarly, a 2009 study in the ISC found a lower percentage of VL cases were detected through ACD than PCD in areas with increased educational activities from NGOs [33]. VL populations already have higher poverty levels and lower educational attainment and literacy compared to India’s national average [6, 8, 34], therefore sustaining vigilant disease identification is especially challenging and crucial in neglected areas. Even where VL cases are infrequent in meso- and low-incidence areas, reinforcing educational aspects of index case-based ACD might facilitate longer-term diagnosis through PCD.

### Blanket & camp ACD in low-incidence districts

Clustered outbreaks have been recently documented in previously low-incidence settings in Bihar, possibly in part due to decreased host competence in areas with few VL infections [35]. In these populations, a greater proportion show decreased immunity over time and are disproportionately susceptible to transmission from acute VL infections [36, 37]. Other studies show a correlation in time and space between outbreaks in previously non-endemic areas adjacent to endemic areas, especially in highly impoverished populations [38, 39]. Curtailing such outbreaks may require more robust ACD tactics, where drivers of transmission are related to lack of previous VL exposure, high poverty, and risk-related proximity to new cases [40].

In this, blanket and camp ACD may be best utilised as a deployable intervention in low-incidence areas at risk of VL outbreaks. Although blanket and camp ACD unit costs were eight times higher than index case-based ACD, it remains a valid strategy for two reasons: 1) it targeted high-incidence areas and may actually be more beneficial for low-incidence areas, and 2) the value of detecting additional cases early may be significant enough to support the high initial investment necessary for reaching EPHP.

Focusing house-to-house surveillance on districts with less than 200 cases per year could be more valuable than presuming it will capture a high number of cases in high-incidence areas. Detecting few cases in low-endemic areas would likely yield the same high unit cost but at a greater epidemiological advantage. Blanket ACD might also be strategically implemented around months of the year when VL transmission is greatest.

### Horizontal programme integration

To date, VL treatment and prevention programmes have been predominantly vertical in India. Given the importance of continued surveillance, yet the high cost of identifying cases at the village level, it will be prudent to eventually integrate VL ACD horizontally with other infectious disease programmes. VL blanket and camp ACD tactics align with other skin- and fever-related disease surveillance strategies, both of which are ongoing in the ISC. Several recent studies document feasibility of integrating VL into ACD for febrile illnesses such as malaria, tuberculosis, and leprosy, particularly using the camp approach [41, 42].

Additionally, historical data trends show a spike in VL transmission often occurs every 15–17 years, which will likely be exacerbated by relaxed control measures as incidence is lowered [43, 44]. Even when EPHP benchmarks are reached, it will be important to sustain VL surveillance in some form to avoid and prepare for potential transmission resurgence. The appropriate stage for integration, additional resources required, and potential risk of neglecting cases due to decreased concentration on VL should be explored.

To support ACD scale-up in the immediate, it may be best to focus on the cost of additional tests conducted rather than additional cases detected. Figs 9 and 10 show the same investment is needed when relying on the blanket and camp strategy for 50% of additional tests conducted but only 25% of additional VL-positive cases detected. Although blanket and camp ACD is undeniably more costly, a diversified case-finding strategy may be critical around this period of EPHP. ACD scale-up should ideally be strategised and coordinated by one entity, such as NVBDCP. As VL incidence further declines, it will become necessary to document all ACD outputs at the block and village level, which the index case-based programme must adopt.

Costs and outcomes reported in this study may be applicable to VL strategies on the ISC, or other diseases aiming toward elimination where active case finding is increasingly relied on. It is imperative that ACD tactics, outputs, and barriers be readily shared between programmes in India to continue supporting a timeline and best strategy for achieving EPHP thresholds. Future research on the investment needed to reach VL EPHP should include expenditures and outcomes of other control activities such as indoor residual spraying (IRS) for sand fly vector control, treatment to remove the threat of PKDL as a reservoir, and antigen diagnostic tests.

## Conclusion

The characterisation and projection of VL case detection costs are fundamental to India’s elimination strategy. As VL cases become both more difficult and critical to find, this study provides insight into how PCD and index case-based ACD might be enhanced by targeting blanket and camp ACD in areas with lower incidence. This analysis should be built upon in future economic studies, particularly where horizontal ACD programme integration is considered. Blanket and camp ACD must be investigated for its additional value to addressing PKDL, co-infections, and marginalised groups. Economic evaluations should also be integrated into complementary disciplines that forecast the impact of case finding strategies alongside the likelihood of reaching elimination, such as mathematical modelling. Cost analyses provide compelling information for decision makers to strategise resource allocation and programme activities and must be encouraged and expanded as NTD-endemic countries approach disease elimination.

### Limitations

This study includes several limitations. First, it is possible that some cases reported in each distinct ACD or PCD activity overlap between programmes. No patient identifiers were collected in this study, where future research may consider tracing suspected cases from village-level identification to confirmed treatment.

Uncertainty may also exist in the generalisation of costs across different populations, given varying VL endemicity and the potential for co-infection with other diseases. This study was limited in availability of block- and village-level data, which would have more accurately informed programme outputs within specific incidence levels.

Bias may be present in reported expenditures of each programme, especially under- or over- estimation of inputs and time spent on activities. To address this, staff-time and input allocation was estimated through micro-costing the hours, resources, and frequency of activities in each strategy. Determining micro-costing parameters relied on interviews and direct observation, and therefore may include bias of self-reporting by staff or in generalising observed activities across other facilities. However, the diversity of data sources, lack of missing data, and sensitivity analyses should provide a best estimate regarding overall inputs, costs, and outputs of each strategy.

This study did not include costs of treatment of VL cases in India, under the assumption that all VL cases identified at the PHC-level are treated the same day. There may have been loss to follow up between VL-positives identified through ACD and those cases confirmed at PHCs. Loss to follow-up in index case-based ACD was addressed by documenting and tracing potential cases between ACD officers, doctors at adjacent PHCs, and NVBDCP. In a similar attempt to minimize loss to follow-up, confirmed cases found through blanket and camp ACD were given formal documentation of their VL-positive test result, allowing them to proceed directly to treatment at the PHC.

## Supporting information

S1 Appendix Cost model assumptions and sources. Assumptions, data sources, and literature references informing the cost model for VL ACD and PCD in Bihar, India during 2018.
(DOCX)
