# Supplementary material for: Costs and outcomes of active and passive case detection for visceral leishmaniasis (Kala-Azar) to inform elimination strategies in Bihar, India
Source: PLoS Negl Trop Dis. 2021 Feb 3;15(2):e0009129. doi: 10.1371/journal.pntd.0009129 (PMC7886142; doi:10.1371/journal.pntd.0009129)
Supplement: S1 Appendix — Assumptions, data sources, and literature references informing the cost model for VL ACD and PCD in Bihar, India during 2018. (DOCX) [file pntd.0009129.s001.docx]

| **VARIABLE** | **ASSUMPTIONS** | **SOURCE** |
| --- | --- | --- |
| ***All Programmes*** | | |
| Cost per VL test | 350INR or $4.75USD (+/- 50% in the sensitivity analysis) | rK-39 manufacturers  (Bio-Rad Laboratories, InBios International Inc, CTK Biotech Inc)  and VL literature |
| Discount Rate | 3% (0%, 6% in the sensitivity analysis) | World Bank Inflation Rates |
| Treatment (excluded) | All patients are eventually diagnosed (or re-confirmed VL-positive) at the hospital (PHC) level and immediately treated with AmBisome. The cost for AmBisome treatment would be consisted in all three programmes, therefore cost of treatment was excluded to focus on cost of diagnosis and case identification. | National Vector Borne Disease Control Programme (NVBDCP) Guidelines (2017) |
| ***Index Case-Based ACD*** | | |
| Economic life years of start-up | 3 years was determined the average start-up period to roll out CARE’s ACD programme and get the KAMIS database off the ground. Start-up included mostly training and travel costs. | CARE records and staff interviews |
| Outputs | Total number of patients suspected, tested, and found VL-positive through CARE’s ACD programme were recorded by DPOs, KBCs, and lab technicians and ultimately recorded in the KAMIS database. This analysis relied on data reported in KAMIS, but triangulated average monthly patient tests and positives by interviewing DPOs, KBCs and lab technicians. | KAMIS and staff interviews |
| Training | Conference venue rates included subsistence (650INR or $8.81USD per person), and 18% tax added, plus rental of podium, projector, and microphone  CARE estimated all travel costs were included in staff salary for training, therefore this study included travel expenses as economic costs (not declared by the provider) | CARE records and staff interviews |
| Vehicles | CARE claimed transportation vehicles (motorbikes) of Kala-Azar Block Coordinators as a stipulation of their hiring. Therefore, CARE did not include this expense as a financial cost, and this study included the cost of each motorbike as an economic cost. The cost of an average motorbike was estimated from direct observation and staff interviews. Manufacturer prices were used to calculate vehicle price, where expected useful life of the bike and average discount rate of capital goods were used to evaluate depreciation. | Staff interviews, direct observation, and manufacturer prices |
| Salaries | 1. State Programme manager (SPM) and SPM Jr. (1,200,000INR or $16,271USD per year at 5% allocation) 2. Field Research Officer (960,000INR or $13,017USD per year at 50% allocation) 3. District Programme Officer (696,000INR or $9,437USD per year at 50% allocation) 4. Kala-Azar Block Coordinator (240,000INR or $3,254USD per year at 75% allocation) 5. ASHA (550INR or $7.45USD per year at <5% allocation) | CARE records and staff interviews |
| Vehicle Operation | Vehicle and motorbike operating costs (fuel, oil, maintenance, registration, etc) were estimated from both staff interviews and local prices for equivalent services. Again, CARE included this cost in the salary of each staff member, where this analysis included vehicle operating costs as economic costs. | Staff interviews, local (Patna, Bihar) prices for fuel, oil, insurance, maintenance, registration, etc |
| Building Operation | Furnishing, electricity and rent were determined from some CARE records and costs of equivalent office space in Patna and were allocated individually between the three staff members who manage the programme from CARE headquarters (two staff work <5% while another worked 75%). | CARE records and online rental price for equivalent space |
| Training & recurrent training | Accommodation, travel, salary, and subsistence fees per training session were estimated from staff interviews and occurred an average of once per year for DPOs and KBCs. Quality control (or quality assurance) was considered to be a part of recurrent training, and included in these estimations. | CARE records and staff interviews |
| Personnel time allocation | Due to discrepancies in staff interviews between supervisors and fieldworkers on personnel allocation, direct observation was used to triangulate minutes spent on each activity (and +/-20% was used in the sensitivity analysis). Supervisors claimed that ACD officers worked 30% of their time on the programme, while the ACD officers themselves claimed to work between 75-95%. Therefore, time and frequency of activities were estimated from direct observation and used to determine KBCs work 75% and DPOs work 50% on ACD activities. The sensitivity analysis varied this allocation by 20% to better capture the large discrepancy. | Staff interviews and direct observation |
| Supplies | Patient case registers were the main supply expense for each District Programme Officer and Kala-Azar Block Coordinator, with a useful life of one year. | Staff interviews and direct observation |
| Equipment | Cell phones were the main equipment used by KBCs and DPOs, and the average cost was estimated from the current retail value in 2019 and converted to the base year of 2018. | Staff interviews, direct observation, and manufacturer prices |
| Waste Management | Not disclosed to investigator and may be negligible | No record in CARE |
| ***Blanket & Camp ACD*** | | |
| Economic life years of start-up | Initial training costs were sourced from KalaCORE’s expenses dataset from 2017, detailing costs in the start-up up year of 2015 and lasted 2 years. Costs were annualized and translated into the base year 2018. Transport costs detail flights from Delhi to Patna for Project managers (reported in lump-sum) and local travel for managers within Patna to conduct training. Trainers present and total number of field coordinators and ASHAS (participants) were estimated through interviews and the data expenses spreadsheet. Financial salary costs were reported for FCs to attend this training, which included travel and per diem. Accommodation was detailed separately. Cost of training venue was not reported, therefore the cost of hiring an equivalent venue for three days (reported training time) was estimated in Patna, India and included as an economic cost.  The start-up period is defined as the inception period, where one month in 2015 was dedicated to developing training protocol and plans prior to the first ACD round implementation. Management fees (salaries) were estimated from the inception report detailing phase 1 ACD monthly, where project staff were shown to work 1 month before the start of the program. Economic costs of ASHAs taking one day off from normal work to attend training are included. | KalaCORE expenditure reports and staff interviews |
| Outputs | Project outputs included number of people screened, number of people tested for VL, and total VL positive cases during 2018. No cases are included in the start-up period. Although KalaCORE detailed patient data down to the village level (including district, block, and village where patient was screened, tested, and diagnosed), data was only considered on the district level to match data in the CARE dataset. | KalaCORE programme report |
| Salary | Both staff salary per grade and allocation factors detailed in KalaCORE expenditure reports | KalaCORE expenditure reports |
| Vehicle operation | For managerial staff, only one vehicle was purchased for the programme, and the remainder of vehicles were rented vehicles or taxis. These costs were included as a lump sum (specific to staff position) in the travel cost section. Travel costs were triangulated by estimating distance and fuel using Google maps.  For fieldworkers, vehicle operation and maintenance costs were detailed in the expenditure spreadsheet as lump sums for fuel, oil, maintenance, registration, and spare parts. These costs reported in KalaCORE’s expenditure report were triangulated with staff interviews. | KalaCORE expenditure reports and staff interviews |
| Capital goods | Building and storage costs were detailed in KalaCORE’s assets register from 2015-2018. Office costs were minimal and only included 1 table and 4 chairs. Other capital costs included additional equipment used in the KalaCORE office in Patna (laptop, microwave, refrigerator). | KalaCORE expenditure reports, asset registers, and manufacturer prices |
| Camp operation | Economic costs of using local health facilities (sub-centres) for diagnostic camps at the end of each week were included, as the centres were dedicated to VL testing rather than normal operations. The sub-centre’s normal cost of operation and equipment use was estimated by referring to government reports on construction, size, equipment, electricity, and supplies lists. The allocation factor for use of this space for one day was estimated using information from staff interviews on the utility of each item for the diagnostic camp. The cost of operating a sub-centre for one day was estimated by referring to another costing study conducted in Norther India during 2014 (and costs were translated to the base year of 2018). | KalaCORE expenditure reports, staff interviews, and IPE Global interviews |
| Retraining | Recurrent training was recorded as a lump sum per staff level (fieldworkers, ASHAs, or additional refresher trainings). | KalaCORE expenditure reports and staff interviews |
| Waste management | Waste management was estimated by protocol detailed by project managers during interviews, along with medical waste disposal costs reported for Bihar by government facilities. rK-39 test disposal occurred after each camp was held, with waste transferred back to Patna for disposal at a government health facility. Travel costs for waste disposal were included in the staff’s ‘overall travel costs’, but was evaluated as an economic cost as an activity funded by NVBDCP (and not KalaCORE). | Staff interviews and KalaCORE expenditure reports |
| Recurrent costs | Other recurrent costs included management fees or goods and services taxes, detailed in KalaCORE’s expenditure dataset. Management fees are attributable to consultancy from IPE Global, which was confirmed during interviews with KalaCORE project managers. | KalaCORE expenditure reports and staff interviews |
| ***Passive Case Detection*** | | |
| Economic life years of start-up | Although India’s Government of Health and Family Welfare was the provider in this programme’s cost analysis, KalaCORE was responsible for implementing the new VL treatment programme in PHCs over 2 years, including medical officer training, ice-lined refrigerators for cold chain storage, and rK-39 and AmBisome distribution to each facility. KalaCORE’s asset registers, programme expenditures, and interviews with staff detailed the start-up costs for passive case detection, and were recorded as economic costs (not financial) as they were funded by DFID and not the Government of India | KalaCORE staff interviews and expenditure reports |
| Salaries | Doctor, lab technician, and nurse salaries were estimated from average local salaries published on governmental public records for similar positions in Bihar, which were also triangulated through staff interviews. A generalization was made (based on several interviews) that the average hospital has one medical officer, one auxiliary nurse, and two lab technicians per VL-unit that assist in patient intake and diagnosis. Salaries were varied by 10% in the sensitivity analysis. | Local job forums and staff interviews |
| Training | Staff trainings were conducted by KalaCORE and MSF during AmBisome rollout (the start-up period). Training costs estimated frequency, participation, and standard operating procedures of initial trainings that targeted doctors, nurses and lab technicians. Training was considered part of their ‘continuing medical education’ and staff were paid their normal salary, but this study took into account the economic cost of staff not engaging in normal working activities. No additional compensation for travel or subsistence was given. Retraining occurred 1-2 days per year and involved one trainer from MSF or KalaCORE during 2018. | KalaCORE records and interviews with medical staff, lab technicians, and trainers from IPE Global (contracted by KalaCORE) |
| rK-39 tests | rK-39 costs were estimated using distributor prices online, and included as an economic costs as they are donated goods and not paid for by the provider. | Manufacturer prices (Bio-Rad Laboratories, InBios International Inc, CTK Biotech Inc) |
| Equipment | Medical equipment costs were drawn from KalaCORE reports detailing cost of ILR, diagnostic tests, and various administrative supplies (desks, chairs, fans, refrigerators). Other capital costs in patient rooms were documented through observation (average room included two beds, desk, fan, ILR, small fridge/freezer, patient registrars, phone, waste disposal) and estimated through distributer prices in Bihar online, and converted to the base year of 2018. A 50% allocation was estimated for diagnostic use of equipment, as the other 50% was excluded as dedicated for treatment (according to staff interviews, direct observation, and standard operating procedures published by NVBDCP). | National Vector Borne Disease Control Programme (NVBDCP) published equipment lists, staff interviews and direct observation |
| Supplies | Recurrent supplies include rK-39, rK-16 diagnostics, gloves, and other general medical supplies (such as disinfectant, disposable goods, and administrative supplies) were documented through staff interviews, direct observation, and average diagnostic materials for vector-borne disease units published in the literature. Costs were estimated using manufacturer pricing online, alongside KalaCORE expenditure reports. The cost of parasitological diagnosis through bone marrow and spleen aspiration were estimated from a previous costing study (Boelaert 1999) and converted to 2018 base year costs for the 17% of patients that could not be diagnosed with a Rapid Diagnostic antibody test (according to KAMIS database). | Staff interviews, direct observation, standard operating procedures (NVBDCP), and literature (Boelaert 1999) |
| Building, storage, and patient overhead | Size of patient rooms were estimated through direct observation (3 square meters), collated with government PHC reports of operational capacity, and averaged across all 73 hospitals were ILRs and AmBisome had been distributed as part of the national Kala-Azar Elimination Programme. Annual rent in Bihar was estimated to be 30INR or $0.41USD per square foot during 2018 according to rental information in equivalent spaces. Patient allocation was estimated to be 50% for diagnosis and 50% for treatment, the former of which was used in the cost model. Waste management was also documented within patient overhead costs. | Direct observation, staff interviews, and costing literature detailing VL patient diagnostic costs within hospitals in Northern India (Chatterjee 2013) |
| Economic life years of capital | Life years of capital were estimated from literature specific to Northern India and staff interviews, with an average working life of 20 years for general office equipment. The working life of medical equipment (ILR, lab equipment, and diagnostic tests) was estimated from each individual manufacturer. | Staff interviews and average life span of medical supplies in the literature (specific to Northern India) |
| Personnel time allocation | Time spent on patient intake and diagnostic activities was self-reported by medical officers and triangulated with standard operating procedures for each rK-39 test, bone marrow aspiration procedure, laboratory activities, and general patient administrative activities. | Staff interviews, direct observation, standard operating procedures, administration, and average outpatient time in hospital from the literature |
| Quality Control (monitoring) | Two vehicles were dedicated to Monitoring and Evaluation officers who oversaw ILRs, rK-39 stock and use, and medical officer training. Costs of these two M&E officers were considered economic (as funded by KalaCORE and not the provider), where supplies, equipment, salaries and travel costs were included in the overall PCD cost. | KalaCORE expenditure reports and staff interviews |
| Outputs | Number of VL cases screened, diagnosed, and treated were estimated by the lab technician and patient registry. Number of patients diagnosed in hospitals during 2018 was estimated using the KAMIS database. The number of VL cases documented by HMIS in Bihar during 2018 was 3,659, where KAMIS documented 3,611. The KAMIS database is assumed to be more reliable in this study, but the sensitivity analysis took into account this slight discrepancy by varying the number of tests conducted by +/- 10%. | Patient registries, KAMIS, HMIS database, and staff interviews |
